# Nurses’ beliefs in the care of newborns at the end of life in the neonatal intensive care unit*

**DOI:** 10.1590/1980-220X-REEUSP-2024-0065en

**Published:** 2024-10-28

**Authors:** Taís de Abreu Ferro, Lucas Thiago Pereira da Silva, Fernanda Machado Silva-Rodrigues, Maiara Rodrigues dos Santos, Regina Szylit

**Affiliations:** 1 Faculdade de Ciências Médicas da Santa Casa de São Paulo, São Paulo, SP, Brazil.; 2 Universidade de São Paulo, Escola de Enfermagem, Programa Interunidades de Doutoramento em Enfermagem, São Paulo, SP, Brazil.; 3 Universidade de São Paulo, Escola de Enfermagem, Departamento de Enfermagem Materno Infantil e Psiquiátrica, São Paulo, SP, Brazil.

**Keywords:** Intensive Care Units, Neonatal, Perinatal Death, Attitude to Death, Health Belief Model, Nurses, Unidades de Cuidado Intensivo Neonatal, Muerte Perinatal, Actitud Frente a la Muerte, Modelo de Creencias sobre la Salud, Enfermeras y Enfermeros

## Abstract

**Objective:**

To describe nurses’ beliefs and attitudes related to care during the end-of-life process and death in a neonatal intensive care unit.

**Method:**

Descriptive and qualitative study with nurses working in a neonatal intensive care unit who experienced care for newborns who died in these units. Data collection was carried out through recorded interviews that were analyzed following thematic analysis from the perspective of the Health Belief Model.

**Results:**

Nurses’ beliefs were categorized in relation to death, nursing care, and perceptions about newborns. The influence of these beliefs on behaviors and care practices was denoted, with the need for emotional support and specific training to manage these situations being highlighted.

**Conclusion:**

Knowledge of the beliefs described in the study is essential to develop more sensitive and comprehensive care strategies, contributing to improve the quality of care in neonatal intensive care units.

## RESUMEN

**Objetivo:**

Describir las creencias y actitudes de los enfermeros relacionados con el cuidado durante el proceso de final de la vida y muerte en una unidad de cuidados intensivos neonatales.

**Método:**

Estudio descriptivo y cualitativo con enfermeras que trabajan en una unidad de cuidados intensivos neonatales y que tuvieron experiencia en el cuidado de recién nacidos fallecidos en dichas unidades. La recolección de datos se realizó a través de entrevistas grabadas y analizadas siguiendo un análisis temático desde la perspectiva del Modelo de Creencias.

**Resultados:**

Se categorizaron las creencias de las enfermeras en relación a la muerte, los cuidados de enfermería y las percepciones sobre los recién nacidos. Fue evidente la influencia de estas creencias en las conductas y prácticas de cuidado, destacándose la necesidad de apoyo emocional y formación específica para el manejo de estas situaciones.

**Conclusión:**

El conocimiento de las creencias descritas en el estudio es fundamental para desarrollar estrategias de atención más sensibles e integrales, contribuyendo a mejorar la calidad de la atención en las unidades de cuidados intensivos neonatales.

## INTRODUCTION

The end of life in the neonatal context profoundly challenges nursing professionals, as the loss of a newborn disrupts the understanding of the natural cycle of life, motivating the search for deeper reasons and meanings^(1)^. In Neonatal Intensive Care Units (NICU), nurses regularly face the death of patients, including those in the first moments of life^(2)^. Statistics show that 75% of neonatal deaths occur in the first week of life, with prematurity, asphyxia, neonatal infections, and congenital anomalies being the main causes observed^(3)^. These deaths, predominantly in hospital settings, expose nurses to emotionally charged situations and ethical challenges.

During professional education, a curative model predominates, which hinders the management of end-of-life situations and provision of care during this period. End of life care comprises the phase in which the health condition indicates a continuous and inevitable decline towards death, with individualized assistance plans^(4)^ being required. In neonatal ICU settings, decisions about this type of care raise complex issues that affect not only the child and their family, but also the health team and the professional directly involved in care^(5)^. The death of the patient seems to invalidate the care provided, and the limit of involvement becomes a dilemma to be faced by the nursing team^(6)^.

The literature on the subject indicates that neonatal nurses experience different attitudes and emotions, from grief to exhaustion when faced with pediatric death^(6,7)^. The challenge for these nurses is to consider newborns as fragile and vulnerable individuals, requiring close relationships with families^(8)^. This proximity can result in positive attitudes, such as the inclusion of the family in the care of the patient at the end of life, or initiatives to participate in care after death. However, negative attitudes may arise, such as frustration at losing patients despite efforts^(9)^. Nurses face burnout, stress, and moral distress when caring for terminally ill newborns^(6,10)^, dealing with distress and fatigue when caring for babies in the context of complex chronic diseases, especially when the risk of death is imminent^(11,12)^.

It is known that personal beliefs significantly influence professionals’ decisions and behaviors^(13)^. Beliefs, in this context, represent perceptions of individuals and groups about the world around them, reflected in actions and behaviors^(13,14,15)^. Such beliefs, whether limiting or facilitating, have the potential to decrease or increase, respectively, people’s abilities to face everyday challenges, intensifying or minimizing suffering, and originate from numerous lenses, including sociocultural issues and subjectivities, related to their life experiences, age, religion, and even the lack or not of dialogue about death during education^(14)^. Beliefs guide behaviors and interpretations. In this regard, they can be limiting, requiring exploration and change; or facilitative, deserving recognition and strengthening^(15)^. Initially used in research with families facing serious illnesses, this model has been applied to support care decisions by nursing professionals^(16,17)^.

Given the above, it is crucial to understand the beliefs of nurses in neonatal ICUs and their influence on care in end-of-life situations. From this perspective, this study aimed to describe nurses’ beliefs and attitudes related to care during the end-of-life process and death in a neonatal intensive care unit.

## METHOD

### Design of Study

Descriptive study with a qualitative approach, developed with nurses working in a neonatal ICU. The detailed presentation of the information in this research was guided and structured by COREQ (Consolidated Criteria for Reporting Qualitative Research)^(18,19)^.

### Local

The interviews took place according to the nurses’ preferences. Three interviews were conducted at the participants’ homes in São Paulo, two at the workplace, four in public places, and three online. In all situations, we sought to ensure privacy and comfort for the participants. Among the participants there were professionals from public and private sectors from different Brazilian hospitals.

### Population and Selection Criteria

Nurses who met the following inclusion criteria were invited to participate in the study: having had the experience of caring for newborns who died in a neonatal ICU and having worked in this sector for at least one year. Those who were on sick or maternity leave during the data collection period were excluded. The technique used to recruit participants was *snowball sampling* ^(20)^. The first contacts established included nurses from the researchers’ contact network and from the research group Interdisciplinary Nucleus for Research in Loss and Bereavement (NIPPEL), registered with the CNPq (National Council for Scientific and Technological Development), of which the authors of this study are part.

During recruitment, there were four refusals. The first occurred in the initial approach of one of the nurses, who was concerned about the possibility that her participation could be perceived as an evaluation of her performance in caring for newborns in serious conditions. Two refusals were due to the potential discomfort that participation in the study could cause. The last one was due to the lack of response to the invitation, without an explicit justification.

### Data Collection

Initially, upon receiving indications from potential participants, researchers contacted them by phone or via messaging app, introducing themselves, indicating who had recommended them, and explaining the objectives of the interview. Once the participant accepted participating voluntarily, the interview was scheduled. At the face-to-face meeting, the consent form was presented for signature and a demographic questionnaire was applied, followed by the main questions: “Do you remember the last baby you cared for as it was dying?” “What was it like taking care of him/her in the neonatal ICU?” “What is it like to care for a baby who dies in the neonatal ICU?” The questions were previously tested to ensure semantic coherence. Given the changes in the questions clarity, this interview was not considered for data analysis.

Data collection took place from July to November 2018 and July to August 2021.The second moment aimed to validate beliefs with new participants. All interviews at this stage were remote, with no comments or corrections. New participants were indicated by previous participants, receiving an introductory email, reason for contact, research objectives and consent form. The interviews were conducted by two researchers in training, supervised by an experienced researcher. The interviews were recorded digitally for later transcription.

The average interview time was 43 minutes. Field notes were made regarding contextual details that could contribute to the triangulation of data sources and to the interpretations of transcribed interviews.

### Data Analysis and Treatment

The interviews were analyzed following thematic analysis^(21)^, according to the steps: (1) Familiarization with the data; (2) Initial coding; (3) Search for themes; (4) Review of themes; (5) Definition and naming of themes; (6) Production of the report. Finally, data were organized into three main themes, which present the dimensions of nurses’ beliefs, described in the subthemes. The interpretation was guided by the chosen theoretical framework, considering beliefs as facilitators and limiters of care^(15)^. This approach allowed a deep understanding of the meaning of each belief and how nurses perceive the experience of caring for a newborn who dies in the neonatal ICU, from the perspective of the health belief model.

### Ethical Aspects

All recommendations of Resolution 466/12 of the National Health Council, which regulates research involving human beings, were followed. The project was submitted for approval by the Ethics and Research Committee of the USP School of Nursing, under opinion number 2.759.147/2018. Ethical precautions were taken when approaching participants, with the signing of the Free and Informed Consent Form taking place prior to the start of the research, whether it took place in person or remotely, and space was provided for questions regarding the process of participation in the research. In order to preserve the confidentiality of participants, their statements are represented by the letter E followed by the number indicating their order of participation in the interviews (1, 2, 3...).

The full content of the interviews is available in the open access repository SciELO data at: https://doi.org/10.48331/scielodata.MYIXKC.

## RESULTS

Twelve professionals participated in the study, all female, aged between 25 and 48 years. The time since graduation ranged from 2 to 21 years and the period of work in neonatal ICU ranged from 2 to 21 years as well; however, the average number of years of experience in the sector was relatively greater (11.2 years) than the average number of years of higher education (9.5 years), as two interviewees reported that they had already worked, before their higher education, as nursing technicians in neonatal ICU.

As mentioned above, the analysis of the data obtained allowed identifying eleven categories, which were organized into three dimensions that represent the diversity of beliefs related to the care of newborns who die in the neonatal ICU.

**Figure 1:**
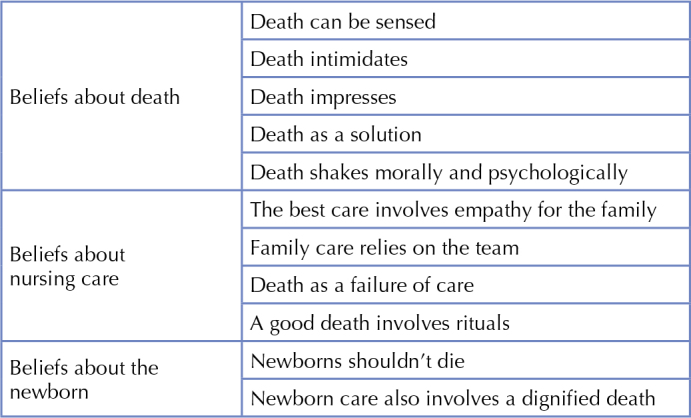
Beliefs and dimensions of care for newborns at the end of life in the Neonatal Intensive Unit. Sao Paulo, Brazil, 2022.

The following chart organizes beliefs into their respective dimensions (Figure 1):

### Beliefs About the Death of a Newborn

Beliefs about the death of a newborn reflect dimensions related to the finitude of newborns in the ICU. In this dimension, five beliefs were observed that portray the nurses’ relationship with death in the neonatal ICU: *death can be sensed; death intimidates; death impresses; death as a solution; death shakes morally and psychologically*.

*Death can be sensed* represents the nurses’ belief that it is possible to identify the proximity of the newborn’s death.

*The clinical profile of the baby who dies, it kind of prepares us, you know? Those of us who have a little more experience, we know that this is a baby that will progress to death. (E3)*

This belief is mainly related to prognoses of extremely premature newborns or those with severe malformations that seriously compromise their clinical condition. Anticipated or expected death influences the behavior of nurses in relation to the care provided to newborns in the days preceding their death. The belief that death can be sensed seemed to facilitate the nurses’ decision to approach the newborn and parents.

In the perspective that *death intimidates*, nurses recognize the profound impact the death of a newborn has on everyone in the NICU environment. They note that death not only profoundly affects healthcare professionals, but also generates fear and anxiety among family members present. The ICU is often perceived as a space of tension, where the possibility of death generates distrust and fear. This perception of death as something that causes fear and concern leads professionals to adopt a posture of concealment, isolating newborns with no prospect of survival.

*These babies were kept in the last care bay, where there was a monitor because it was mandatory, for us to be able to confirm the time of death, and they were left there, without any assistance, they didn’t have their veins punctured, they were not fed, nothing. They stayed there quietly with monitoring until the time of death. (E2)*

*Death impresses* was represented by the belief that death awakens a natural feeling of compassion for loss. It represents the physical and emotional shock that affects the team after the announcement of the death, highlighting the need for space and time to reorganize emotions.

*At that moment when he told me that the baby had died, I went to the bathroom and said, “Wow, I need 5 minutes.” You know those 5 minutes? I kept thinking, I thought a little, I cried a little, but then I came back... (E8)*

The belief that death impresses still motivates nurses to seek ways to alleviate their own suffering.

*Death as a solution* was the belief that reflected the meaning given by nurses to experiencing death, especially of newborns with neurological impairments or syndromes that impact the quality of life of the baby and the family. The nurses reported that invasive procedures, which can lead to the deterioration of the newborn’s clinical or physical condition and generate permanent after-effects, end up making them reframe death, which is seen as a relief for the suffering of everyone involved.

*Watching him die wasn’t the hard part. Knowing that he was dying, that he wasn’t going to come back from the arrest, that he really wasn’t going to respond because he was no longer able, wasn’t the hard part. The complicated thing is knowing that it didn’t have to be that way. And even if he had died from the underlying pathology, he didn’t need to go through all that... (E7)*

This view of death as a solution seemed to help deal with the feelings associated with the loss of the newborn in the ICU.

*Death shakes morally and psychologically* represented the set of thoughts and feelings experienced by nurses after each death in the neonatal ICU, especially those that were sudden or unexpected. When experiencing the sudden deaths of newborns who appeared clinically stable, sometimes even expected to be discharged, nurses questioned the possibility that they had failed in some assessment or intervention carried out with these babies.

*There is a lot of expectation that the baby will overcome this problem, then suddenly the baby presents a change, the arrest comes and the baby doesn’t come back... these are cases that shake me the most. Because progression is right in front of our eyes [...] and suddenly, right? It takes us by surprise. These are the ones that shock me the most.* (E11)

The sudden death of a newborn in the neonatal ICU is emotionally devastating for the healthcare team. This experience challenges the team’s core mission of ensuring patient survival and creates a sense of loss of control over care. It requires a period of reorganization and acceptance of the inevitability of death, despite the strong bonds formed with the newborn’s family. The following statement illustrates this complex emotional reality:

*You are overcome by a desire to save, to make people live [...] it was horrible, everyone was very shocked. Death is always shocking, it is always sad, it is always bad, it is always… Even more so when you create a bond, when the child has been there for a long time and you become attached. It is impossible for you not to become attached to the family and the child. (E2)*

### Beliefs About Nursing Care

This dimension consisted of the nurses’ beliefs regarding nursing care for newborns at the end of life in the neonatal ICU. These beliefs were shaped by the nurses’ experiences, based on their observations, what they heard and their feelings in situations of death in this context. They reflect the accumulated interaction with the team, the family and the newborn over time. Among the beliefs identified, key aspects emerged that describe nursing care from the participants’ perspective, namely: *The best care involves empathy for the family; family care depends on the team; death as a failure of care; a good death involves rituals*.

*The best care involves empathy for the family* concerns nurses’ belief that it is not possible to provide the best care without embracing, approaching, or considering the family in end-of-life care. Nurses believe that when facing the death of a newborn in the ICU, the family must be present during their final moments and after their death. They seek to bring the family closer to the dying baby, allowing visits, enabling parents to hold their still-living children during the process and maintain contact with the body after death. Thus, the belief in empathic care for the family transforms the approach to the newborn, redefining care centered on family empathy.

*The best care I gave to a baby who died, I think, was to place it in its mother’s arms. I think you need to give it to the family, you know? [...] We see that it is leaving? Give it to the mom, you know? It’s hers! The child is not mine, it is not the doctor’s, it is theirs, you know? It’s for the family, let them say goodbye with dignity, with love, it’s their moment.” (E2)*

The *family care depends on the team* – addresses the belief that the care model centered on the child and family needs to be part of end-of-life care; however, this is only viable when the team agrees with this practice. It includes the belief in the need to inform and embrace the family, requiring adherence from the interdisciplinary team. Nurses believe that family care in the face of newborn death benefits from an interdisciplinary approach. Grief for the family and newborn persists; however, the provision of care varies depending on the team in the NICU.

*With this baby, I kept saying, “Doctor, are you going to call the mother? Are you going to call the father?” Because the doctor was so focused [on the clinical worsening] that he forgot a bit about that part. Don’t mom and dad want to pick it up? Let a grandfather in, let an aunt in, a godmother in. I once had a situation where the mother said, “Can’t a friend of mine come in?” “No, she can’t! “But she is the baby’s godmother.” So she’s not just a friend! There’s a bond involved there. (E5)*

The belief of nurses in *death as failure of care* reflects their perception of the role of nursing and its responsibility in this process. They expressed concern that they may have missed signs or made mistakes that could have led to the newborn’s death. This belief, fueled by fear of imperfections in care and the view that preserving life is the primary goal, reinforces the notion that death can be a direct result of failures in care.

*Sometimes I do question myself, if there were any mistakes... Of not premeditating, of not thinking about something. Did the baby give us some sign that we didn’t notice? Was there no measure to prevent this from happening? What if we had given some drugs before? Antibiotics before? If we had tests done before? So I always take a look at the medical record to investigate, to see to what extent the team was correct and what they missed. (E9)*

The results also highlighted that *a good death involves rituals*. Nurses recognize the need to respect families’ diverse cultures and religions, even if they are different from their own. They emphasize the value of the family’s religious vision in the moments before and after death. According to nurses, care during the dying process or at the time of death transcends simple physical assistance to the body, involving symbolic elements that are significant for different cultures.

*Because you offer religious support. That’s what she [the grandmother] wanted! I don’t know the gypsy religion, whether what she’s saying is true or not is not up to me. She is saying that the baby needs to spend this time in someone’s company to open this path of light and that’s it. So let’s do it for her. (E5)*

### Beliefs About the Newborn

This dimension represents the beliefs arising from the relationship between death and the target audience for nursing care: *Newborns shouldn’t die* and *newborn care also involves a dignified death*.

*Newborns shouldn’t die* represents the nurses’ belief that, being at the beginning of life, their death under medical care seems incongruent. This view clashes with the professional ideal of saving lives, making it difficult to accept serious complications or malformations in these babies.

*It was my first job and, when a premature baby, who had a malformation, died, the doctor said there was nothing more that could be done, and the baby died. I thought: “How come? The baby dies?” When he told me there was nothing more to do, I looked at him and asked myself: “What do you mean, you’re not going to try anymore?” (E8)*

Furthermore, this belief makes it difficult to develop strategies that help give new meaning to the concept of ‘good care’ in situations of loss.

*Newborn care involves providing a dignified death*, according to the nurses’ belief. This means keeping the baby in a comfortable and private environment, minimizing pain and discomfort when death is inevitable. Nurses note that the focus of care is often on the illness and the family, while the newborn receives less attention. They are dedicated to presenting the baby in an honorable manner to the family, including actions such as bathing the baby respectfully and dressing the baby in meaningful clothing. For nurses, this care represents a gesture of respect for the newborn under their care.

*I chose R’s clothes. I asked his mother that day... “If something happens, what clothes do you want me to put on him?” And she said, “I’d like you to put on this jumpsuit...” He died, I put on that jumpsuit, I put his favorite toy, I prepared the body with his favorite toy, I took him to the morgue with his favorite toy… (E4)*

## DISCUSSION

The main objective of this study was to explore nurses’ beliefs related to care during the end-of-life process and death in the neonatal ICU. A variety of beliefs related to the end-of-life process of newborns was found, organized into three dimensions: beliefs about death, beliefs about nursing care, and beliefs about the newborn.

When we deal with beliefs, it is important to consider that they are a lens through which we look at the world and, therefore, knowing them is only possible through interaction with others and the analysis of the influence of biopsychosocial aspects^(15)^. It was observed that demographic factors such as sex, level of education, frequency of contact with newborns at the end of life, and experience in neonatal care at the end of life, presented similarities with the findings of research conducted in Greece^(22)^, with health professionals in the area of neonatology, mostly women, with less than 20 years of professional experience and more than 5 years in neonatal ICU, as in our study.

When it comes to beliefs related to nursing care, the authors of this same study were able to relate the impact of neonatal deaths in the NICU on the personal lives of nurses who frequently avoided thoughts and conversations about death, correlating them negatively with fear and anxiety. This corroborates aspects identified in our study, which also reveals the predominance of beliefs that represent barriers to care rather than facilitating elements.

Our beliefs distinguish us from other people, but they also bring people together and influence the beliefs of others^(15)^. Nevertheless, death is commonly viewed as an event to be avoided or postponed, creating the perception that the process of dying is in direct opposition to the act of living. Personal values and beliefs serve as fundamental strategies that assist professionals in approaching death. The death of patients can trigger both personal and professional effects for the entire team involved^(22)^. Understanding the systems of meaning that shape professionals’ actions when facing death is crucial, impacting the quality of care.

In Türkiye, a study with a similar methodological design interviewed 7 neonatal ICU nurses. Nurses expressed sadness and anguish over the loss of patients, questioning their actions and feeling guilty, highlighting their commitment to keeping babies alive, reflecting on their actions and seeking answers after deaths^(23)^.

Our results revealed divergences among participants regarding pro-life attitudes, in line with a cross-sectional study involving 279 neonatal nurses in Jordan^(24)^. This study showed that nurses generally believe in the need for efforts to ensure the newborn survival, even in serious situations. Similarly, Brazilian nurses in our study expressed a strong inclination toward preserving life. However, some also considered the implications of survival in cases of severe sequelae or circumstances that significantly compromise the babies’ quality of life. It should be noted that certain beliefs are more acceptable in some cultures and in certain contexts. The relevance of certain beliefs, or lack thereof, depends on the judgment of observers and the origin of those beliefs^(15)^.

Nurses who base their decisions on the perspective of quality of life are more likely to seek a dignified death^(24)^. This dignity is not limited to the choice of not prolonging futile treatments, but also encompasses empathy with the family’s suffering and respect for funeral rites, even when they differ from those practiced by the team. Studies^(6,24)^ indicate that nursing professionals tend to emphasize the importance of their values and beliefs when participating in decisions related to the end of life. Although this aspect was mentioned by some participants in our study, no details about religious and individual beliefs were provided.

A particularly notable aspect is the nurses’ perception of the possibility of anticipating the newborn’s death, which influences their attitudes. Although this concept is not widely explored in other recent studies, some of them recognize the importance of a realistic assessment of the unfavorable prognosis of the newborn and the need to establish limits in intensive care. Furthermore, these studies, like ours, highlight the role of personal influences on health professionals. They show how nurses balance considerations about death and the quality of life of the newborn with the belief in the sanctity of human life, which implies the valorization of each life, regardless of its health status or prognosis^(6,23,24)^.

The statements reveal the active participation of the interviewees in end-of-life decisions, expressing opinions on the continuity of treatment and offering support to the family, valuing the newborn’s quality of life. A recent study highlighted the importance of nurses’ participation in these issues. In it, Greek nurses showed an inclination to preserve life or adopt a quality of life-centered approach, influenced by personal factors such as experience of motherhood/fatherhood and daily practice in the NICU^(6)^.

In our study, a striking observation is the concept of dignified death for newborns in neonatal ICUs. Nurses believe that, in the face of inevitable death, it is essential to provide a care environment that respects the newborn’s integrity and dignity. This involves carefully managing physical symptoms, such as pain and discomfort, and respecting the emotional and spiritual aspects of end-of-life. Practices include creating a calm and comfortable environment, sensitively involving the family, and performing rituals that honor the baby’s brief but meaningful life. This focus on dignified death reflects a holistic approach that recognizes the newborn as a complete human being, whose life, however short, deserves respect and care^(25,26)^.

Current literature highlights advances in discussions about nurses’ experiences in end-of-life care for newborns. The significant influence that these situations have on the personal lives and careers of these professionals is recognized. Beliefs are truths of a subjective reality that influence biopsychosocial functioning^(15)^. Therefore, it is crucial to develop a detailed understanding of nurses’ attitudes and beliefs, which are decisive in their actions and approaches. Understanding these aspects is vital to developing effective teaching strategies aimed at identifying and adjusting pre-existing beliefs, reinforcing more sensitive and appropriate care practices. This study makes a valuable contribution by deeply exploring these crucial aspects of health care.

As with any research, especially on sensitive and little-explored topics, this study faces limitations. Among them, the limited scope of the public stands out, which, despite being suitable for qualitative analyses, does not reflect the country’s cultural diversity. The variability in the interviewees’ training time may also have influenced their perspectives. Despite this, the results presented here contribute to the understanding of the professionals’ beliefs and attitudes in a sensitive and complex care scenario.

## CONCLUSION

This study provides an in-depth and multifaceted insight into the beliefs and attitudes of neonatal ICU nurses regarding end-of-life care for newborns.. Such beliefs were organized by their relationship with the dimensions of death, nursing care, and newborn care. Its results describe a systematization of beliefs that can facilitate the recognition of these dimensions as precursors of attitudes and behaviors in the care of newborns in ICUs. Professionals seek strategies to deal with loss, recognizing family suffering, and strive to embrace the family by offering a comfortable environment, respecting their rituals. Despite sadness and anguish, the nurses felt they had fulfilled their duties in providing the best care possible to the newborn and the family. These findings indicate the need for emotional support and specific training for nurses to manage these situations.

The results indicate the need to expand spaces with multidisciplinary groups to discuss the newborns’ care at the end of life, providing a safe and collaborative environment to share experiences and emotional challenges. These findings may be a catalyst for future research and dialogue on neonatal care in end-of-life situations, to enrich understanding of the topic and promote more compassionate and holistic approaches. Incorporating these insights into care policies and practices can substantially improve the quality of support provided to newborns and their families during the most challenging times.
