# Supplementary material for: Nurses’ beliefs in the care of newborns at the end of life in the neonatal intensive care unit
Source: Rev Esc Enferm USP. 2024 Oct 28;58:e20240065. doi: 10.1590/1980-220X-REEUSP-2024-0065en (PMC11534307; doi:10.1590/1980-220X-REEUSP-2024-0065en)
Supplement: Translation

# Translation: Crenças de enfermeiras no cuidado de recém-nascidos em final de vida na unidade intensiva neonatal*

**DOI:** 10.1590/1980-220X-REEUSP-2024-0065pt

**Authors:** Taís de Abreu Ferro, Lucas Thiago Pereira da Silva, Fernanda Machado Silva-Rodrigues, Maiara Rodrigues dos Santos, Regina Szylit

**Affiliations:** 1 Faculdade de Ciências Médicas da Santa Casa de São Paulo, São Paulo, SP, Brasil.; 2 Universidade de São Paulo, Escola de Enfermagem, Programa Interunidades de Doutoramento em Enfermagem, São Paulo, SP, Brasil.; 3 Universidade de São Paulo, Escola de Enfermagem, Departamento de Enfermagem Materno Infantil e Psiquiátrica, São Paulo, SP, Brasil.

**Keywords:** Unidades de Terapia Intensiva Neonatal, Morte Perinatal, Atitude Frente a Morte, Modelo de Crenças de Saúde, Enfermeiras e Enfermeiros

## Abstract

**Objetivo:**

Descrever as crenças e as atitudes das enfermeiras relacionadas ao cuidado durante o processo de fim de vida e a morte em unidade de terapia intensiva neonatal.

**Método:**

Estudo descritivo e qualitativo com enfermeiras atuantes em unidade de terapia intensiva neonatal que vivenciaram a experiência de cuidar de recém-nascidos que morreram nessas unidades. A coleta de dados foi realizada por meio de entrevistas gravadas e analisadas seguindo a análise temática sob a perspectiva do Modelo de Crenças.

**Resultados:**

As crenças das enfermeiras foram categorizadas em relação à morte, cuidados de enfermagem e percepções sobre os recém-nascidos. Evidenciou-se a influência dessas crenças nos comportamentos e práticas de cuidado, destacando-se a necessidade de apoio emocional e formação específica para o manejo dessas situações.

**Conclusão:**

O conhecimento das crenças descritas no estudo é fundamental para desenvolver estratégias de cuidado mais sensíveis e abrangentes, contribuindo para a melhoria da qualidade da assistência em unidades de terapia intensiva neonatal.

## INTRODUÇÃO

O fim de vida no contexto neonatal desafia profundamente os profissionais de enfermagem, pois a perda de um recém-nascido perturba a compreensão do ciclo natural da vida, motivando a busca por razões e significados mais profundos^(1)^. Nas Unidades de Terapia Intensiva (UTI) Neonatal, enfermeiros enfrentam regularmente a morte de pacientes, inclusive nos primeiros momentos de vida^(2)^. Estatísticas apontam que 75% das mortes neonatais ocorrem na primeira semana de vida, sendo a prematuridade, asfixia, infecções neonatais e anomalias congênitas as principais causas observadas^(3)^. Essas mortes, predominantemente em ambientes hospitalares, expõem enfermeiros a situações emocionalmente carregadas e a desafios éticos.

Na formação profissional, predomina um modelo curativo, que dificulta o manejo de situações de fim de vida e a provisão dos cuidados nesse período. Cuidados de fim de vida compreendem a fase em que a condição de saúde indica um declínio contínuo e inevitável em direção à morte, sendo necessário o planejamento e implementação de planos assistenciais individualizados^(4)^. Nos ambientes de UTI neonatal, as decisões desses cuidados levantam questões complexas que afetam não apenas a criança e sua família, mas também a equipe de saúde e o profissional diretamente envolvido na assistência^(5)^. O óbito do paciente parece invalidar o cuidado prestado, e o limite do envolvimento torna-se um dilema a ser enfrentado pela equipe de enfermagem^(6)^.

A literatura sobre o tema aponta que enfermeiras neonatais experienciam diversas atitudes e emoções, desde o luto até a exaustão diante da morte pediátrica^(6,7)^. O desafio para essas enfermeiras é considerar os recém-nascidos como indivíduos frágeis e vulneráveis, exigindo relações próximas com as famílias^(8)^. Esta proximidade pode resultar em atitudes positivas, como a inclusão da família nos cuidados ao paciente em fim de vida, ou iniciativas de participação nos cuidados após a morte. No entanto, podem surgir atitudes negativas, como a frustração pela perda de pacientes apesar dos esforços^(9)^. As enfermeiras enfrentam esgotamento, estresse e sofrimento moral ao cuidar de recém-nascidos em fim de vida^(6,10)^, lidando com angústia e fadiga ao cuidar de bebês em contextos de doenças crônicas complexas, especialmente quando o risco da morte é iminente^(11,12)^.

Sabe-se que as crenças pessoais influenciam significativamente as decisões e comportamentos dos profissionais^(13)^. Crenças, nesse contexto, representam percepções de indivíduos e grupos sobre o mundo ao seu redor, refletindo em ações e condutas^(13,14,15)^. Tais convicções, limitantes ou facilitadoras, têm o potencial de diminuir ou aumentar, respectivamente, as capacidades das pessoas para enfrentarem os desafios do cotidiano, intensificando ou minimizando o sofrimento, e originam-se de inúmeras lentes, incluindo questões socioculturais e subjetividades, relacionadas às suas experiências de vida, idade, religião e até mesmo à falta do diálogo sobre a morte ou não na formação^(14)^. As crenças orientam comportamentos e interpretações. Nesse sentido, podem ser limitantes, necessitando exploração e alteração; ou facilitadoras, merecendo reconhecimento e fortalecimento^(15)^. Inicialmente empregado em pesquisas com famílias diante de doenças graves, este modelo tem sido aplicado na fundamentação de decisões de cuidado por profissionais de enfermagem^(16,17)^.

Dado o exposto, é crucial compreender as crenças das enfermeiras em UTI neonatal e sua influência no cuidado em situações de fim de vida. Nessa perspectiva, esse estudo objetivou descrever as crenças e as atitudes das enfermeiras relacionadas ao cuidado durante o processo de fim de vida e a morte em unidade de terapia intensiva neonatal.

## MÉTODO

### Tipo do Estudo

Estudo descritivo de abordagem qualitativa, desenvolvido com enfermeiras atuantes em UTI neonatal. A apresentação detalhada das informações desta pesquisa foi norteada e estruturada pelo COREQ (Consolidated Criteria for Reporting Qualitative Research)^(18,19)^.

### Local

As entrevistas ocorreram conforme a preferência das enfermeiras. Três entrevistas foram realizadas na casa das participantes em São Paulo; duas no local de trabalho, quatro em locais públicos e três online. Em todas as situações, buscou-se assegurar privacidade e conforto às participantes. Dentre as participantes havia profissionais de setores públicos e privados de diferentes hospitais brasileiros.

### População e Critérios de Seleção

Foram convidados a participar do estudo enfermeiros que atendessem aos seguintes critérios de inclusão: ter vivenciado a experiência de cuidar de recém-nascidos que morreram em UTI neonatal e trabalhar nesse setor há pelo menos um ano. Excluíram-se aqueles que estivessem de licença médica ou maternidade no período de coleta de dados. Para o recrutamento dos participantes foi utilizada a técnica *snow ball sampling* (amostragem em bola de neve)^(20)^. Os primeiros contatos estabelecidos incluíram enfermeiras da rede de contato dos pesquisadores e do grupo de pesquisa Núcleo Interdisciplinar de Pesquisa em Perdas e Luto (NIPPEL), cadastrado no CNPq, do qual fazem parte os autores deste estudo.

Durante o recrutamento, ocorreram quatro recusas. A primeira ocorreu na abordagem inicial de uma das enfermeiras, que ficou receosa com a possibilidade de que sua participação pudesse ser percebida como uma avaliação do seu desempenho no cuidado a recém-nascidos em estado grave. Duas recusas deram-se devido ao potencial desconforto que a participação no estudo poderia causar. Já a última delas, deu-se pela falta de resposta ao convite, sem uma justificativa explícita.

### Coleta de Dados

Inicialmente, ao receber as indicações dos potenciais participantes, os pesquisadores entravam em contato telefônico ou por aplicativo de mensagens, apresentando-se, indicando quem os havia recomendado e explicando os objetivos da entrevista. Com o aceite do participante para sua participação voluntária, agendava-se a entrevista. No encontro presencial, apresentava-se o termo de consentimento para assinatura e aplicava-se um questionário demográfico, seguido pelas questões principais: “Você se lembra do último bebê que cuidou quando morria?” “Como foi cuidar dele na UTI neonatal?” “Como é cuidar de um bebê que morre na UTI neonatal?”. As questões foram testadas previamente para garantir coerência semântica. Dadas as modificações na clareza das questões, essa entrevista não foi considerada para a análise dos dados.

A coleta de dados ocorreu de julho a novembro de 2018 e julho a agosto de 2021. O segundo momento visou validar as crenças com novos participantes. Todas as entrevistas nessa fase foram remotas, sem comentários ou correções. Os novos participantes foram indicados pelos anteriores, recebendo e-mail de apresentação, motivo do contato, objetivos da pesquisa e termo de consentimento. As entrevistas foram conduzidas por dois pesquisadores em treinamento, supervisionados por uma pesquisadora experiente. As entrevistas foram registradas por gravação digital, para posterior transcrição.

O tempo médio de entrevistas foi de 43 minutos. Notas de campo foram feitas a respeito de detalhes contextuais que pudessem contribuir para a triangulação das fontes de obtenção dos dados e para as interpretações das entrevistas transcritas.

### Análise e Tratamento dos Dados

As entrevistas foram analisadas seguindo a análise temática^(21)^, conforme as etapas: (1) Familiarização com os dados; (2) Codificação inicial; (3) Busca por temas; (4) Revisão dos temas; (5) Definição e nomeação dos temas; (6) Produção do relatório. Ao final, os dados foram organizados em três temas principais, que apresentam as dimensões das crenças dos enfermeiros, descritas nos subtemas. A interpretação foi guiada pelo referencial teórico escolhido, considerando crenças como facilitadoras e limitadoras do cuidado^(15)^. Esta abordagem permitiu uma compreensão profunda do significado de cada crença e como as enfermeiras percebem a experiência de cuidar de um recém-nascido que morre na UTI neonatal, sob a perspectiva do modelo de crenças em saúde.

### Aspectos Éticos

Foram seguidas todas as recomendações da Resolução 466/12 do Conselho Nacional de Saúde, que regulamenta as pesquisas envolvendo seres humanos. O projeto foi submetido à aprovação pelo Comitê de Ética e Pesquisa da Escola de Enfermagem da USP, sob parecer de número 2.759.147/2018. Foram observados os cuidados éticos para a abordagem dos participantes, sendo que a assinatura do Termo de Consentimento Livre e Esclarecido ocorreu previamente ao início da pesquisa, tenha ela ocorrido de maneira presencial ou remota, e propiciou-se espaço para dúvidas a respeito do processo de participação na pesquisa. Com o objetivo de preservar o sigilo dos participantes, suas declarações estão representadas pela letra E seguida pelo número que indica sua ordem de participação nas entrevistas (1, 2, 3...).

O conteúdo das entrevistas na íntegra encontra-se disponível no repositório de acesso aberto SciELO data sob o link: https://doi.org/10.48331/scielodata.MYIXKC.

## RESULTADOS

Participaram do estudo 12 profissionais, todas do sexo feminino, que tinham idade entre 25 e 48 anos. O tempo de formação variou de 2 a 21 anos e o período de atuação em UTI neonatal variou de 2 a 21 anos também, porém, a média de anos de experiência no setor mostrou-se relativamente maior (11,2 anos) do que a de formação superior (9,5 anos), pois duas entrevistadas informaram que já trabalhavam, antes de sua formação superior, como técnicas de enfermagem em UTI neonatal.

Como supracitado, a análise dos dados obtidos possibilitou identificar onze categorias, que foram organizadas em três dimensões que representam a diversidade de crenças relacionadas ao cuidado de recém-nascidos que morrem na UTI neonatal.

O quadro a seguir organiza as crenças em suas respectivas dimensões (Figura 1):

**Figure 1:** Crenças e dimensões do cuidado de recém-nascidos em fim de vida na Unidade Intensiva Neonatal. São Paulo, SP, Brasil, 2020.

### Crenças Sobre a Morte de um Recém-Nascido

As crenças sobre a morte de um RN refletem dimensões relacionadas à finitude de recém-nascidos em UTI. Nessa dimensão foram observadas cinco crenças que retratam a relação das enfermeiras com a morte na UTI neonatal: *a morte pode ser pressentida; a morte intimida; a morte comove; a morte como solução; morte abala moral e psicologicamente*.

A *morte pode ser pressentida* representa a crença das enfermeiras de que é possível identificar a proximidade da morte do RN.

*O perfil do bebê que vai a óbito, ele meio que prepara a gente, sabe? A gente que tem um pouquinho mais de experiência, de vivência, a gente sabe que é um bebê que vai evoluir a óbito. (E3)*

Essa crença está relacionada, principalmente, a prognósticos de recém-nascidos prematuros extremos ou com malformações graves que comprometem gravemente a sua condição clínica. A morte pressentida ou esperada influencia o comportamento das enfermeiras em relação aos cuidados prestados ao recém-nascido nos dias que antecedem a sua morte. A crença de que a morte pode ser pressentida pareceu facilitar a decisão das enfermeiras em se aproximarem do RN e dos pais.

Na perspectiva de que *a morte intimida*, as enfermeiras reconhecem o impacto profundo que a morte de um recém-nascido tem sobre todos no ambiente da UTI neonatal. Elas observam que a morte não apenas afeta profundamente os profissionais de saúde, mas também gera medo e inquietação entre os familiares presentes. A UTI é frequentemente percebida como um espaço de tensão, onde a possibilidade de morte gera desconfiança e receio. Essa percepção da morte como algo que causa medo e inquietação leva os profissionais a adotarem uma postura de ocultação, isolando recém-nascidos sem perspectiva de sobrevivência.

*Esses nenéns ficavam no último box, onde tinha o monitor porque era obrigado, pra poder constatar a hora do óbito e ficavam lá, sem assistência nenhuma, não puncionavam veia, não faziam alimento, nada. Eles ficavam lá quietinhos com a monitorização até a hora do óbito. (E2)*

A *morte comove* foi representada pela crença de que a morte desperta um sentimento natural de compaixão pela perda. Representa o abalo físico e emocional que recai sobre a equipe após o anúncio do óbito, alertando para a necessidade de um espaço e um tempo para reorganizar as emoções.

*Naquele momento que ele falou para mim que o bebê tinha ido a óbito, eu fui ao banheiro eu falei Nossa, eu preciso de 5 minutos” sabe aqueles 5 minutos? Eu fiquei pensando, pensei um pouco, chorei um pouco, mas depois eu voltei... (E8)*

A crença de que a morte comove ainda motiva as enfermeiras em buscar maneiras de aliviar o próprio sofrimento.

A *morte como solução* foi a crença que refletiu o significado dado pelas enfermeiras ao experienciar a morte, especialmente de RN com comprometimentos neurológicos ou síndromes que impactam a qualidade de vida do bebê e da família. As enfermeiras relataram que procedimentos invasivos, que podem levar à deterioração do estado clínico ou físico do recém-nascido e gerarem sequelas permanentes, acabam por fazê-las ressignificar a morte, que passa a ser vista como um alívio para o sofrimento de todos envolvidos.

*Vê-lo morrer não foi a parte difícil. Saber que ele estava morrendo, que ele não ia voltar da parada, que ele realmente não ia responder porque ele não tinha mais condições, não foi a parte difícil. O complicado é você saber que não precisava ter sido daquele jeito. E que mesmo que ele tivesse morrido da patologia de base, ele não precisava ter passado por tudo aquilo… (E7)*

Esta visão da morte como uma solução pareceu ajudar a lidar com os sentimentos associados à perda do recém-nascido na UTI.

A *morte abala moral e psicologicamente* representou o conjunto de pensamentos e sentimentos vividos pelas enfermeiras após cada óbito vivenciado na UTI neonatal, especialmente aqueles repentinos ou inesperados. Ao vivenciarem as mortes repentinas de recém-nascidos que pareciam estáveis clinicamente, por vezes até com previsão de alta, as enfermeiras se questionaram sobre a possibilidade de terem falhado em alguma avaliação ou intervenção realizada com esses bebês.

*Toda uma expectativa para que o bebê vença esse problema, aí de repente o bebê apresenta uma alteração, vem a parada e o bebê não volta… são casos que me abalam mais. Porque está na nossa cara a evolução [...] e de repente, né? Pega a gente de surpresa. São os que me chocam mais.* (E11)

A morte repentina de um recém-nascido na UTI neonatal é emocionalmente devastadora para a equipe de saúde. Essa experiência confronta a missão central da equipe, que é assegurar a sobrevivência do paciente, e provoca uma sensação de perda de controle sobre o cuidado. Ela exige um período de reorganização e aceitação da inevitabilidade da morte, apesar dos fortes laços formados com a família do recém-nascido. A seguinte declaração ilustra essa complexa realidade emocional:

*Você fica tomado por uma coisa de salvar, de fazer viver [...] foi horrível, ficou todo mundo muito chocado. A morte sempre choca, sempre é triste, sempre é ruim, sempre é… Ainda mais assim, quando você cria laço, quando a criança já está lá há muito tempo e você se apega. É impossível você não se apegar à família e à criança. (E2)*

### Crenças Sobre os Cuidados de Enfermagem

Essa dimensão foi composta pelas crenças das enfermeiras em relação aos cuidados de enfermagem de recém-nascidos em fim de vida na UTI neonatal. Estas crenças foram moldadas pelas experiências vivenciadas pelas enfermeiras, baseadas em suas observações, no que ouviram e em seus sentimentos nas situações de morte nesse contexto. Elas refletem a interação acumulada com a equipe, a família e o próprio recém-nascido ao longo do tempo. Dentre as crenças identificadas, emergiram aspectos chave que descrevem os cuidados de enfermagem na perspectiva das participantes, a saber: *O melhor cuidado envolve empatia à família; o cuidado à família depende da equipe; a morte como falha no cuidado; a boa morte envolve rituais*.

O *melhor cuidado envolve empatia à família* diz respeito à crença das enfermeiras de que não é possível oferecer o melhor cuidado sem acolher, aproximar ou considerar a família nos cuidados de fim de vida. Enfermeiras creem que, ao enfrentar a morte de um recém-nascido na UTI, a família deve estar presente durante seus momentos finais e após a sua morte. Buscam aproximar a família do bebê que está morrendo, permitindo visitas, possibilitando que os pais segurem seus filhos ainda vivos durante o processo e mantenham contato com o corpo após a morte. Assim, a crença no cuidado empático à família transforma a abordagem ao recém-nascido, redefinindo o cuidado centrado na empatia familiar.

*O melhor cuidado que eu dei para um bebê que morreu, eu acho que foi colocar no colo da mãe. Acho que precisa dar para a família, entendeu? [...] A gente está vendo que está indo embora? Dá para a mãe, sabe? É dela! A criança não é minha, não é do médico, é deles, sabe? Dá para a família, deixa eles se despedirem com dignidade, com amor, é um momento deles.” (E2)*

O *cuidado à família depende da equipe* – aborda a crença de que o modelo assistencial centrado na criança e na família precisa compor o cuidado de fim de vida; no entanto, o mesmo só é viável quando a equipe está de acordo com esta prática. Inclui a crença na necessidade de informar e acolher a família, requerendo adesão da equipe interdisciplinar. As enfermeiras creem que o cuidado familiar diante da morte do recém-nascido beneficia-se de uma abordagem interdisciplinar. O pesar pela família e pelo recém-nascido persiste; contudo, a oferta de cuidado varia conforme a equipe na UTI.

*Nesse bebê, o tempo todo eu falava assim “Doutor, vai chamar a mãe? Vai chamar o pai?” Porque o médico estava tão focado [na piora clínica] que esquece um pouco dessa parte. Será que mãe e o pai não querem pegar? Deixa um avô entrar, deixar uma tia, uma madrinha. Uma vez eu tive uma situação que a mãe falou “Uma amiga minha não pode entrar?” “Não, não pode! “Mas ela é a madrinha do bebê”. Então não é só uma amiga! Tem um vínculo ali envolvido. (E5)*

A crença das enfermeiras da *morte como falha no cuidado* reflete sua percepção sobre o papel da enfermagem e a sua responsabilidade nesse processo. Elas expressaram preocupação com a possibilidade de terem negligenciado sinais ou cometido erros que poderiam ter levado à morte do recém-nascido. Essa crença, alimentada pelo medo de imperfeições no cuidado e pela visão de que a preservação da vida é o objetivo principal, reforça a noção de que a morte pode ser um resultado direto de falhas no cuidado prestado.

*Às vezes eu me questiono sim, se não houve falhas... De não premeditar, de não estar pensando em alguma coisa. Será que o bebê deu algum sinal que a gente não percebeu? Será que não tinha alguma medida para evitar isso ter acontecido? Será que se a gente tivesse entrado com alguma droga antes? Com antibiótico antes? Se não tivesse feito exames antes? Então eu sempre dou uma olhada no prontuário para investigar, para saber até que ponto a equipe foi correta e o que deixou passar*. (E9)

Os resultados também destacaram que *a boa morte envolve rituais*. As enfermeiras reconhecem a necessidade de respeitar as diversas culturas e religiões das famílias, mesmo que diferentes das suas próprias. Elas enfatizam a valorização da visão religiosa da família nos momentos que antecedem e sucedem a morte. Segundo as enfermeiras, os cuidados durante o processo de morrer ou no momento da morte transcendem a simples assistência física ao corpo, envolvendo elementos simbólicos significativos para diferentes culturas.

*Porque é você oferecer o apoio religioso. Era o que ela [a avó] queria! Eu não conheço a religião dos ciganos, se é verdade ou não o que ela está falando não cabe a mim. Ela está falando que a bebê precisa ficar esse tempo na companhia de alguém para abrir esse caminho de luz e ponto. Então vamos fazer isso para ela. (E5)*

### Crenças Sobre o Recém-Nascido

Esta dimensão representa as crenças originadas pela relação da morte com o público-alvo dos cuidados de enfermagem: *Recém-nascidos não deveriam morrer* e *o cuidado ao recém-nascido também envolve uma morte digna*.

*Recém-nascidos não deveriam morrer* representa a crença das enfermeiras de que, estando no início da vida, sua morte sob cuidados médicos parece incongruente. Essa visão confronta o ideal profissional de salvar vidas, tornando difícil aceitar complicações graves ou as más-formações nesses bebês.

*Foi meu primeiro emprego e, quando ocorreu o óbito de um bebê que era prematuro e tinha má formação, o médico afirmou que não havia mais o que fazer, e o bebê morreu. Eu pensei: “Como assim? Bebê morre?” Ao me dizer que não havia mais o que fazer, eu olhei para ele e questionei internamente: “Mas como assim, não vai tentar mais?”. (E8)*

Além disso, essa crença dificulta o desenvolvimento de estratégias que ajudem a atribuir um novo significado ao conceito de ‘bom cuidado’ em situações de perda.

*O cuidado ao recém-nascido envolve proporcionar uma morte digna*, segundo a crença das enfermeiras. Isso significa manter o bebê em um ambiente confortável e privativo, minimizando dores e desconfortos, quando a morte é inevitável. As enfermeiras observam que, muitas vezes, o foco do cuidado recai sobre a doença e a família, enquanto o recém-nascido recebe menos atenção. Elas se dedicam a apresentar o bebê de maneira honrosa à família, incluindo ações como banhá-lo respeitosamente e vesti-lo com roupas significativas. Para as enfermeiras, esses cuidados representam um gesto de respeito ao recém-nascido sob seus cuidados.

*Eu escolhi a roupa do R. Eu perguntei para a mãe naquele dia... “Se acontecer alguma coisa qual a roupa que você quer que coloque nele?” E ela falou “Eu gostaria que colocasse esse macacão...” Ele foi a óbito, eu coloquei aquele macacão, eu pus o brinquedo dele favorito, eu preparei o corpo com o brinquedo preferido dele, eu o levei para o necrotério com o brinquedo preferido dele… (E4)*

## DISCUSSÃO

O principal objetivo deste estudo foi explorar as crenças das enfermeiras relacionadas ao cuidado durante o processo de fim de vida e a morte em UTI neonatal. Foi encontrada uma variedade de crenças relacionadas com o processo de fim de vida de recém-nascidos, organizadas em três dimensões: crenças sobre a morte, crença sobre o cuidado de enfermagem e crenças sobre o recém-nascido.

Quando tratamos de crenças, é importante considerar que elas são com uma lente para olharmos o mundo e, portanto, conhecê-las só é possível a partir da interação com o outro e da análise da influência de aspectos biopsicossociais^(15)^. Observou-se que fatores demográficos como gênero, nível de escolaridade, frequência de contato com recém-nascidos em fim de vida e experiência em cuidados neonatais em fim de vida, apresentaram similaridades com os achados de pesquisa conduzida na Grécia^(22)^, com profissionais de saúde na área de neonatologia, em sua maioria mulheres, com menos de 20 anos de experiência profissional e mais de 5 anos em UTI neonatal, tal como em nosso estudo.

Em se tratando das crenças relacionadas aos cuidados de enfermagem, os autores desse mesmo estudo conseguiram relacionar o impacto das mortes neonatais na UTIN na vida pessoal das enfermeiras que frequentemente evitavam pensamentos e conversas sobre morte correlacionando-os negativamente com o medo e ansiedade. Isso corrobora aspectos identificados em nosso estudo, que também revela a predominância de crenças que mais representam barreiras no cuidado do que elementos facilitadores.

Nossas crenças nos distinguem das outras pessoas, mas também aproximam pessoas e influenciam as crenças de outros^(15)^. Não obstante, a morte é comumente encarada como um evento a ser evitado ou adiado, criando a percepção de que o processo de morrer está em oposição direta ao ato de viver. Valores e crenças pessoais servem como estratégias fundamentais que auxiliam os profissionais na abordagem da morte. A morte de pacientes pode desencadear efeitos tanto no domínio pessoal quanto profissional para toda a equipe envolvida^(22)^. Compreender os sistemas de significado que moldam as ações dos profissionais durante o enfrentamento da morte é crucial, impactando na qualidade do cuidado.

Na Turquia, uma pesquisa com desenho metodológico semelhante, entrevistou 7 enfermeiras de UTI neonatal. As enfermeiras expressaram tristeza e angústia pela perda de pacientes, questionando suas ações e sentindo-se culpadas, destacando seu empenho em manter os bebês vivos, refletindo sobre suas ações e buscando respostas após os óbitos^(23)^.

Nossos resultados revelaram divergências entre as participantes em relação a atitudes pró-vida, alinhadas com um estudo transversal envolvendo 279 enfermeiras neonatais na Jordânia^(24)^. Este estudo indicou que as enfermeiras geralmente acreditam na necessidade de esforços para garantir a sobrevivência do neonato, mesmo em situações graves. Similarmente, as enfermeiras brasileiras em nosso estudo expressaram forte inclinação à preservação da vida. No entanto, algumas também ponderaram sobre as implicações da sobrevivência em casos de sequelas graves ou circunstâncias que comprometam significativamente a qualidade de vida dos bebês. É válido ressaltar que certas crenças são mais aceitáveis em algumas culturas e em determinados contextos. A pertinência de determinadas crenças, ou a falta dela, depende do julgamento dos observadores e da origem dessas crenças^(15)^.

As enfermeiras que fundamentam suas decisões na perspectiva da qualidade de vida são mais propensas a buscar uma morte digna^(24)^. Essa dignidade não se limita à escolha de não prolongar tratamentos fúteis, abrangendo também a empatia com o sofrimento da família e o respeito aos ritos funerários, mesmo quando diferentes dos praticados pela equipe. Estudos^(6,24)^ indicam que profissionais de enfermagem tendem a enfatizar a importância de seus valores e crenças ao participarem de decisões relacionadas ao final da vida. Embora esse aspecto tenha sido mencionado por algumas participantes de nosso estudo, não foram fornecidos detalhes sobre crenças religiosas e individuais.

Um aspecto particularmente notável é a percepção das enfermeiras sobre a possibilidade de antecipar a morte do recém-nascido, o que influencia as suas atitudes. Embora este conceito não seja amplamente explorado em outros estudos recentes, alguns deles reconhecem a importância de uma avaliação realista do prognóstico desfavorável do recém-nascido e a necessidade de estabelecer limites nos cuidados intensivos. Além disso, estes estudos, assim como o nosso, destacam o papel das influências pessoais dos profissionais de saúde. Eles mostram como as enfermeiras equilibram considerações sobre a morte e a qualidade de vida do neonato com a crença na santidade da vida humana, o que implica na valorização de cada vida, independentemente de seu estado de saúde ou prognóstico^(6,23,24)^.

As declarações revelam a participação ativa das entrevistadas em decisões de fim de vida, expressando opiniões sobre a continuidade do tratamento e oferecendo apoio à família, prezando pela qualidade de vida do recém-nascido. Um estudo recente ressaltou a importância da participação das enfermeiras nessas questões. Nele, enfermeiras gregas mostraram uma inclinação a preservar a vida ou adotar uma abordagem centrada na qualidade de vida, influenciada por fatores pessoais, como experiência de maternidade/paternidade e prática diária na UTIN^(6)^.

Em nosso estudo, uma observação marcante é a concepção de morte digna para o recém-nascido em UTIs neonatais. As enfermeiras acreditam que, diante da morte inevitável, é essencial proporcionar um ambiente de cuidado que respeite a integridade e dignidade do neonato. Isso envolve o manejo cuidadoso de sintomas físicos, como dor e desconforto, e respeito aos aspectos emocionais e espirituais do fim de vida. Práticas incluem criar um ambiente tranquilo e confortável, envolver sensivelmente a família e realizar rituais que honrem a vida breve, porém significativa, do bebê. Este enfoque na morte digna reflete uma abordagem holística que reconhece o recém-nascido como um ser humano completo, cuja vida, mesmo curta, merece respeito e cuidado^(25,26)^.

A literatura atual destaca avanços nas discussões sobre as experiências das enfermeiras no cuidado de fim de vida de recém-nascidos. Reconhece-se a influência marcante que essas situações têm na vida pessoal e na carreira desses profissionais. Crenças são verdades de uma realidade subjetiva que influenciam o funcionamento biopsicossocial^(15)^. Portanto, é crucial desenvolver uma compreensão detalhada das atitudes e crenças das enfermeiras, determinantes em suas ações e abordagens. Compreender esses aspectos é vital para elaborar estratégias de ensino eficazes, destinadas a identificar e ajustar crenças pré-existentes, reforçando práticas de cuidado mais sensíveis e adequadas. Este estudo contribui de maneira valiosa, explorando profundamente esses aspectos cruciais do cuidado em saúde.

Como acontece com qualquer pesquisa, especialmente sobre temas sensíveis e pouco explorados, este estudo enfrenta limitações. Entre elas, destacam-se a abrangência limitada do público, que, apesar de adequada para análises qualitativas, não reflete a diversidade cultural do país. A variabilidade no tempo de formação das entrevistadas também pode ter influenciado as suas perspectivas. Apesar disso, os resultados aqui apresentados contribuem para a compreensão das crenças e atitudes dos profissionais em um cenário de cuidado sensível e complexo.

## CONCLUSÃO

Este estudo oferece uma visão aprofundada e multifacetada sobre as crenças e atitudes de enfermeiras em UTIs neonatais frente aos cuidados no fim de vida de recém-nascidos. Tais crenças foram organizadas por sua relação com as dimensões da morte, com o cuidado de enfermagem e do recém-nascido. Seus resultados descrevem uma sistematização de crenças que podem facilitar o reconhecimento dessas dimensões como precursores de atitudes e comportamentos no cuidado de neonatos em UTIs. As profissionais buscam estratégias para lidar com a perda, reconhecendo o sofrimento familiar, e se esforçam para acolher a família oferecendo um ambiente confortável, respeitando os seus rituais. Apesar da tristeza e angústia, as enfermeiras sentiram o cumprimento de seus deveres ao proporcionar o melhor cuidado possível ao recém-nascido e sua família. Esses achados indicam a necessidade de apoio emocional e formação específica para as enfermeiras gerenciarem estas situações.

Os resultados indicam a necessidade de ampliar espaços com grupos multidisciplinares para discutir os cuidados de neonatos em fim de vida, proporcionando um ambiente seguro e colaborativo para compartilhar experiências e desafios emocionais. Esses achados podem ser um catalisador para futuras pesquisas e diálogos sobre o cuidado neonatal em situações de fim de vida, a fim de enriquecer a compreensão do tema e promover abordagens mais compassivas e holísticas. A incorporação destas percepções nas políticas e práticas de cuidado pode melhorar substancialmente a qualidade do apoio oferecido a neonatos e suas famílias durante os momentos mais desafiadores.
